# Evaluation of Extracellular Matrix Remodeling in Full-thickness Skin Grafts in Mice

**DOI:** 10.1369/00221554231225995

**Published:** 2024-01-24

**Authors:** Anton Erik Tjust, Urban Hellman, Antonios Giannopoulos, Annika Winsnes, Karin Strigård, Ulf Gunnarsson

**Affiliations:** Department of Medical Sciences, Clinical Neurophysiology, Uppsala University, Uppsala, Sweden, Umeå University, Umeå, Sweden; Department of Clinical Sciences, Umeå University, Umeå, Sweden; Department of Public Health and Clinical Medicine, Umeå University, Umeå, Sweden; Surgery, Department of Surgical and Perioperative Sciences, Umeå University, Umeå, Sweden; Surgery, Department of Surgical and Perioperative Sciences, Umeå University, Umeå, Sweden; Surgery, Department of Surgical and Perioperative Sciences, Umeå University, Umeå, Sweden; Surgery, Department of Surgical and Perioperative Sciences, Umeå University, Umeå, Sweden

**Keywords:** collagen, extracellular matrix, fibroblasts, hernia, hyaluronan, mouse model, S100A4, vimentin

## Abstract

Abdominal hernia is a protruding weakness in the abdominal wall. It affects abdominal strength and life quality and can lead to complications due to intestinal entrapment. Autologous full-thickness skin graft (FTSG) has recently become an alternative material for reinforcement in the surgical repair of large abdominal hernias instead of synthetic mesh. FTSG eventually integrates with the abdominal wall, but the long-term fate of the graft itself is not fully understood. This has implications as to how these grafts should be optimally used and handled intraoperatively. This study investigates the remodeling of FTSG in either the onlay or the intraperitoneal position 8 weeks after FTSG transplantation in an experimental mouse model. There was a significant presence of fibroblasts, indicated by vimentin and S100A4 staining, but there were significant variations among animals as to how much of the graft had been remodeled into dense connective tissue. This correlated significantly with the proportion of vimentin-positive cells in the dense connective tissue. We also found that collagen hybridizing peptide staining intensity, a marker of active remodeling, was significantly associated with the proportion of S100A4-positive cells in the dense connective tissue of the FTSG.

## Introduction

Abdominal hernia is a protrusion of abdominal content through the abdominal wall due to a congenital or acquired weakness. The pathophysiology includes changes in connective tissue composition and metabolism.^1^ At present, surgical repair is the only way to restore structural integrity of the abdominal wall. Use of a synthetic or biological prosthetic material considerably reduces risk for hernia recurrence.^2^ The use of full-thickness skin graft (FTSG) in hernia repair has gained popularity in recent years as a more tissue-compatible material with similar or lower complication rates than synthetic alternatives.^3,4^ The three common implant positions in ventral hernia repair are onlay and sublay placement (in relation to the fascia of the external abdominal oblique muscle), and the intraperitoneal (IPOM) position. The choice of placement depends on previous surgery and specific hernia properties.^5^

Our knowledge is sparse on the mechanisms un-derlying tissue remodeling and integration of FTSG implanted in the abdominal wall. Adverse short- and long-term side effects from synthetic implants as well as increasing demands from patients wishing to avoid foreign material have highlighted the need for a greater understanding of the biological implications of the use of FTSG as reinforcement in different anatomical positions. One example is the use of FTSG in the IPOM position, where otherwise a synthetic mesh would come into direct contact with the intestines and other intra-abdominal organs. This is of paramount importance regarding the long-term safety of this technique. Until now, however, FTSG in the IPOM position has only been used in a small pilot series, as well as an ongoing randomized controlled trial on the repair of parastomal hernia.^3,6^

We understand that FTSG undergoes shape adjustment after implantation, merging with the surrounding tissues, but the mechanisms involved in its successful integration remain unknown. Animal models involving skin graft implantation have shown significant matrix remodeling in the graft.^7^ Specific cell types, mainly fibroblasts, facilitate the remodeling process by producing collagen molecules before being arranged into specific configurations. However, this class of cells, despite being the main contributor to the extracellular matrix (ECM) of connective tissues, has not been studied in the context of FTSG implantation. Indeed, fibroblasts have been shown to be much more diverse than previously thought, with multiple lineages within tissues that may respond differently during tissue remodeling and repair. In skin, this is exemplified with the presence of reticular and papillary fibroblasts that have different embryonical origins and respond with different propensities for scar formation during healing.^8^

Classic fibroblast-associated proteins such as S100A4 and vimentin have been shown to promote collagen production in the skin.^9,10^ These markers are also expressed on myofibroblasts that are characterized by increased contractility and ECM production, thus being essential regulators in tissue repair. Furthermore, skin wound-healing studies of skin have shown that vimentin has a complex role in fibroblast function, involving both proliferation, fibroblast activation, and collagen synthesis.^10^ S100A4 has in turn been shown to be a potent activator of fibroblasts in transforming growth factor (TGF)-β-induced fibroblasts, leading to increased synthesis of collagens.^11^ Collagen deposition and arrangement in FTSG are required for integration of the graft into the abdominal wall if it is to withstand the mechanical forces present and prevent hernia recurrence. However, the involvement of S100A4- or vimentin-positive fibroblasts in FTSG integration is unknown. Evaluation of these cell markers in relation to the remodeling process may reveal relevant information on the integration process, and thereby give us valuable information before the widespread introduction of FTSG in hernia surgery.

The skin contains more hyaluronan (HA) than any other connective tissue. HA molecules are negatively charged, attracting water and providing tissues with moisture, which is important for optimal skin function. HA has also been suggested to have a crucial role in tissue regeneration and remodeling processes. Fetal wounds are known for their scarless healing, and this has been associated with the prolonged preservation of HA in the ECM. Furthermore, exogenous HA administration has been shown to promote tissue repair in rodent skin and tendons by regulating collagen turnover, thus contributing to remodeling of the ECM.^12^

The activation of fibroblasts is accompanied by the release of active TGF-β, an important inducer of collagen production, ECM remodeling, and fibrosis.^13,14^ These cells have been shown by us and others to enhance HA synthesis in cell culture.^15,16^ The interaction of HA and the receptor HA-mediated motility receptor causes increased infiltration of fibroblasts that affects collagen production and fibrosis.^17^

Despite being one of the major components in the ECM, almost no scientific description of the role of HA in hernia formation and tissue remodeling after hernia repair has been published. The contribution of HA to the integration of FTSG after hernia surgery is also unknown and could be a key regulator of graft integration, given its early involvement in the cascade of ECM remodeling.

The aim of this study was to evaluate FTSG remodeling and integration processes in settings applicable to human hernia surgery, thereby providing crucial information on the use of FTSG in different positions and in the repair of different hernia types. We used a histological approach using FTSGs obtained from an established murine model to assess structural and regional characteristics of the integrated grafts compared with the presence and abundance of fibroblasts and other markers of ECM remodeling.

## Materials and Methods

### Tissue Preparation

C57BL/6 mice had a FTSG implanted the IPOM or onlay position (*n*=20), as described previously.^18^ Eight weeks after surgery, a complete section of the anterior abdominal wall, including the skin, muscles, peritoneum, and the FTSG, was excised with a scalpel and cut into smaller pieces for fixation in paraformaldehyde before paraffin embedding. Paraffin-embedded blocks with cross sections of the anterior abdominal wall were sectioned at 5 µm at room temperature using a sliding myotome. Sections were collected on a heated water bath and transferred to adhesion microscope slides (Superfrost Plus, Erie Scientific Co; Portsmouth, New Hampshire) and stored for later staining.

## Histology

### Staining for HA With HA-binding Probe (HABP)

The sections were deparaffinated and rehydrated and thereafter incubated in 3% H_2_O_2_ in methanol for 5 min. After washing in distilled water and phosphate-buffered saline (PBS), the sections were incubated with bovine serum albumin (BSA; 10 mg/ml) for 30 min to block nonspecific binding sites. The sections were incubated at 4C overnight with a biotin-marked HABP (1:40 dilution). The following day, the sections were incubated with Vectastain-Elite Avidin-Biotin complex reagent (Vector Laboratories; Burlingame, California) for 40 min, and after washing incubated for 5 min in a solution of 3,3′-diaminobenzidine (Vector Laboratories; Oxfordshire, UK). Nuclear counterstaining with Mayer’s hematoxylin was followed by dehydration and mounting on cover slips.

### Masson Trichrome Staining

The sections were deparaffinated, rehydrated, and fixed in Bouin’s solution overnight. After washing in distilled water, the cell nuclei were stained with Weigert’s hematoxylin for 5 min. Biebrich Scarlet-Acid Fushin for 5 min stained the cytoplasm, muscles, and collagen. Collagen was decolorized for 5 min with phosphotungstenphosphomolbydenic acid solution from the Masson Trichrome stain kit (Sigma-Aldrich; Oakville, Canada). Collagen was stained for 5 min with aniline blue, followed by 2 min in 1% acetic acid. Finally, the sections were dehydrated and mounted on cover slips.

### Immunohistochemistry

Immunohistochemistry labeling was carried out with the following antibodies: α-smooth muscle actin (ab32575, Abcam; Cambridge, UK), S100A4 (ab197896, Abcam), vimentin (ab92547, Abcam), and Ki-67 (14-5698-82, Invitrogen; Waltham, Massachusetts). Deparaffinization was carried out as follows: Slides were pre-heated for at least 20 min in an oven set at 60C and inspected to ensure that all remaining paraffin had liquefied. Heated slides were then immersed in Xylene three times for 5 min with new Xylene changes every 5 min. Sections were then rehydrated through immersion in pure ethanol for 3 min and then 95% ethanol for another 3 min. Deparaffinized rehydrated slides underwent heat-mediated antigen retrieval with pH 6 citrate buffer (Merck; Darmstadt, Germany) for vimentin staining or pH 8 Tris-ethylenediaminetetraacetic acid buffer (Merck) for S100A4 staining. Buffer-immersed slides were heated in a 70C water bath for 30 min and then rinsed three times for 5 min with PBS with 0.05% added Tween20 (Merck; hereafter referred to as PBST) at room temperature. To block nonspecific binding, sections were covered in 400 µl of 5% Goat normal serum (ab7481, Abcam), diluted by volume with PBST and incubated for 15 min at room temperature. Blocking solution was subsequently drained and the primary antibody (Table 1), diluted in PBST with 1% bovine serum, was applied to each section for overnight incubation at 4C. Sections were kept in a leveled moisture chamber to hinder evaporation of solutions from the sections. The following day, sections were returned to room temperature and rinsed three times for 5 min in PBST, followed by 15 min blocking at room temperature with Goat normal serum as above. A secondary antibody (Table 1) was applied for 30 min at 37C. Finally, the slides were rinsed with PBS before mounting on cover slips and a VECTASHIELD antifade media containing 4′,6-diamidino-2-phenylindole (DAPI; Vector Laboratories, Newark, California). In a subset of nine specimens, a Ki-67 primary antibody was added together with the rabbit polyclonal vimentin primary antibody to create a solution with two different primary antibodies. Later, at the step where the secondary antibody is added, a solution of Alexa 488 goat anti-mouse together with Alexa 594 goat anti-rabbit secondary antibodies was used to double-stain for vimentin and Ki-67 simultaneously. Negative primary and secondary antibody controls consisted of slides processed as above, including the application of normal serum and secondary antibodies, but modifying the protocol by (1) omitting the primary antibodies outlined above and just adding secondary antibodies or (2) replacing the primary antibody with a concentration-matched polyclonal rabbit IgG isotype control (ab37415, Abcam). In negative controls without rabbit IgG, labeled cells with fibroblast-like appearance could not be seen in neither the host skin nor the graft. In primary antibody controls with nonspecific IgG rabbit antibodies, the sections showed some nonspecific reactivity with both acidic and alkaline pre-treatment. This nonspecific IgG binding was primarily seen in parts of the FTSG with disrupted keratin and degenerating hair follicles. A nonspecific reactivity could be seen in a minor subset of cells in the connective tissue of both normal skin and the collagenous part of the FTSG. This nonspecific reactivity was seen in <2% of all cells in both parts of the section and was deemed to be of minor impact for the quantification of cells identified with the anti-vimentin and anti-S100A4 rabbit IgG antibodies. Positive controls consisted of sections from abdominal skin from either animals that had not been operated (external positive control) or areas of skin further removed from where the incision was made (internal positive control). In these positive controls, smooth muscle actin, vimentin, and S100A4 antibodies labeled the cytoplasm of cells with stellate fibroblast-like appearance in the dermis of the skin. No sections were considered for further analysis unless these labeled cells were present and clearly stained in the dermis of the section.

**Table 1.** Antibodies used in the study.

Antibody	Supplier	Type	Concentration	Pretreatment/Miscellaneous
Anti-vimentin	Abcam	Rabbit monoclonal	1:300	Citrate pH 6 for 30 min at 70C
Anti-S100A4	Abcam	Rabbit monoclonal	1:500	Tris-EDTA pH 8 for 30 min at 70C
Anti-Ki 67	Invitrogen	Rat monoclonal	1:500	Citrate pH 6 for 30 min at 70C
B-CHP	3Helix	—	1:20	PBS 1% BSA
Rabbit IgG isotype control	Abcam	Rabbit polyclonal	1:300–1:500	Citrate pH 6 for 30 min at 70C or Tris-EDTA pH 8 for 30 min at 70C
Goat anti-rabbit Alexa Flour 594	Abcam	Goat monoclonal conjugated	1:500	Secondary antibody
Goat anti-rat Alexa Flour 488	Abcam	Goat monoclonal conjugated	1:500	Secondary antibody
Alexa Flour 647 Streptavidin	Thermo Scientific	—	1:200	Secondary antibody

Abbreviations: CHP, collagen hybridizing peptide; EDTA, ethylenediaminetetraacetic acid.

### Collagen Hybridizing Peptide (CHP)

CHP staining was performed according to manufacturer’s protocol (3Helix; Salt Lake City, Utah) after the embedding material was removed as described above. Briefly, a working amount of the biotin-conjugated CHP stock solution (0.3 mg/ml at 4C) was diluted in PBS containing 1% BSA to a final concentration of 15 μg/ml and thermally dissociated to single strands by heating for 6 min at 80C. To prevent thermally induced tissue damage, the heated solution was briefly placed in ice. Within 2 min after cooling, the CHP solution was applied to the tissue samples for 2 hr at 4C. The slides were then washed three times for 5 min in PBS, and an Alexa Flour 647 streptavidin conjugate antibody (Thermo Scientific; Waltham, Massachusetts) was added at a concentration of 0.05 mg/ml for 1 hr at room temperature. The slides were then washed three times for 5 min in PBS, and a mounting media with DAPI was applied before mounting on cover slips.

### Imaging Acquisition and Analyses

Slides were scanned with PANNORAMIC 250 Flash III (3DHISTECH, Budapest, Hungary) using the associated software Pannoramic Scanner Software v3.03. The scanned slides were evaluated in SlideViewer v2.5 (3DHISTECH). Evaluation of HA staining was performed by three independent assessors (authors A.E.T., A.G., and U.H.). The dense connective tissue (DCT) of the graft (see the Results section) was localized according to HTX and eosin staining and Masson Trichrome staining, and evaluated using the SlideViewer program according to the following grading protocol: HABP DCT (−) indicating no HABP in the DCT of the graft; HABP DCT (+/−) indicating a speckled appearance where less than half of the fascicles/strands in the DCT were positive for HABP; HABP DCT (+) where 50–75% of the DCT were positive for HABP; and HABP DCT (++) where HABP staining in the DCT encompassed >75% of the fascicles/strands.

Graft cross-sectional area (CSA) and areas of different tissue types were estimated with the inbuilt measurement tools provided with the software. Figures were assembled and annotated in Adobe Photoshop v23.5 (Adobe; San Jose, California).

The CHP intensity units were measured by evaluating three areas per section of normal skin or graft using ImageJ (64-bit; v.2.9.0/1.53t; Java 1.8.0_322). The intensity units were calculated by normalizing the mean intensity with the background intensity.

### Quantification of Vimentin-positive and S100A4-positive Cells

Cross sections of the host skin were identified on each section stained with the abovementioned markers. Regions of 1091 × 682 µm^2^ were preselected along the length of the skin section, omitting areas with section folding, excessive tearing, and where the skin overlay the graft itself. One or two well-preserved areas were randomly selected for quantification. Our goal was to select two well-preserved areas from each specimen but in some cases this was not possible due to excessive tearing of the section. The dermis of the skin was delineated with an area tool, leaving out epidermis, subcutis, and hair follicles in each preselected area. This was done to achieve a quantitative estimate that mirrored the properties of the dermis. The number of DAPI-positive cells was quantified in the area, followed by a second quantification of those DAPI-positive cells that were positive for vimentin or S100A4. Between 34 and 335 cells were counted in each specimen. The total skin area quantified in each specimen was 19,912–258,263 µm^2^. In the areas of the section encompassing the graft, only areas containing DCT were quantified. DCT was identified by simultaneous reviewing of HTX and Masson Trichrome staining on corresponding serial sections. Areas of DCT were identified by their high collagen content and fibrillar structure (see the Results section for details). For all grafts studied, approximately four areas 1091 × 682 µm (as with normal skin) were preselected that contained at least 25% DCT. One or two areas were randomly selected for quantification. Quantification was carried out similarly to that for the dermis in the host animal skin, except that in the DCT there were no hair follicles and visible subcutis to be removed from the region of interest. The total area of DCT was delineated in each region and the cells counted using a counting frame for unbiased quantification. In the graft, 106–804 cells were counted, encompassing 25,820–1,050,932 µm^2^, and the proportion of vimentin- and S100A4-positive cells was calculated, using the total number of DAPI cells counted in the skin and the graft, respectively. The density of vimentin- and S100A4-positive cells was calculated with the number of cells counted per total area of areas analyzed, and reported as positive cells/1000 µm^2^.

### Statistics

Analyses were carried out using the software SPSS 28.0 (IBM; Armonk, New York). Normality of data variables was graphically evaluated with Q–Q plots. Differences between groups (only IPOM and onlay in this study) were assessed with independent *t*-tests for variables with normal distributions, and with Mann–Whitney *U* test for variables where normality could not be assumed. For paired tests, paired *t*-tests were used in normally distributed data and Wilcoxon signed-rank test in data where normality could not be assumed. In analysis of bivariate correlations, Spearman’s rank correlation coefficient was used in all correlations except those bivariate correlations that concerned HABP staining intensity evaluations. Here, Kendall’s tau coefficient (which treats tied ranks more correctly) was used instead. Missing data due to loss of sections from tearing were treated in a pairwise exclusion manner, for example, if one single measurement was missing from one mouse, that did not preclude it from inclusion in correlational testing for other measurements. Therefore, the number of mice included could be less than 20 for some statistical tests. For HABP evaluations, inter-rater congruence was evaluated with Fleiss’ kappa. Generation of bar graphs was carried out in Graphpad prism (Graphpad software; Boston, Massachusetts).

For correlations and differences, a *p* value <0.05 was considered significant.

## Results

### General Morphology

**Figure 1.**
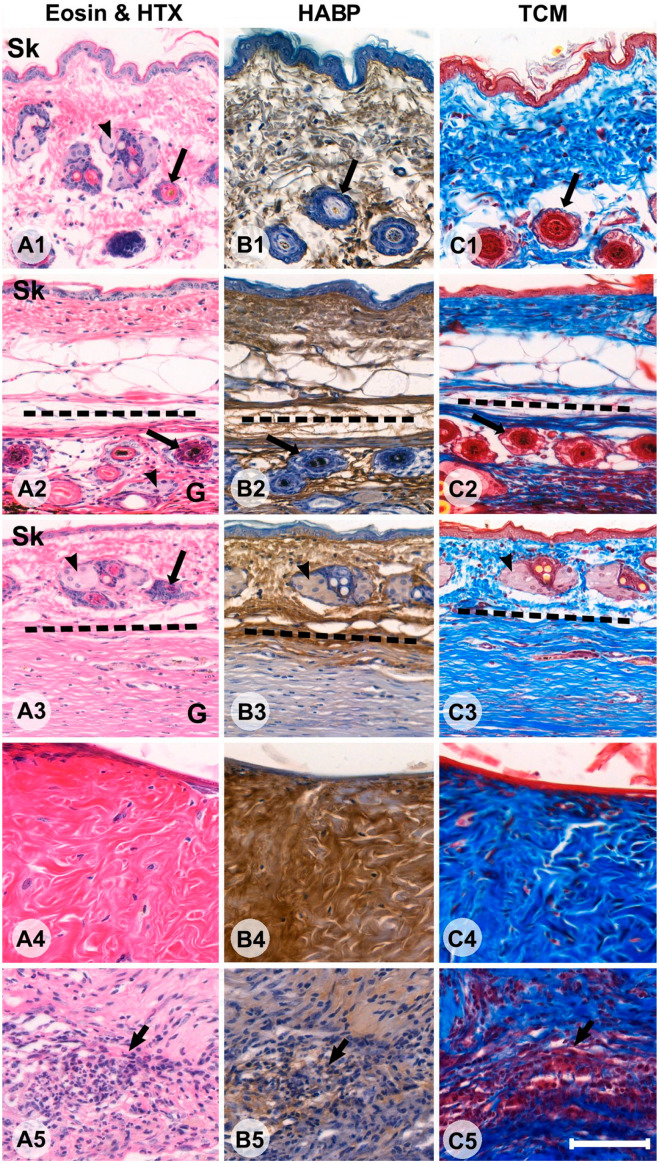
Sections of different specimens showing cross sections of host mouse skin (A1–C1), full-thickness skin graft (FTSG) with limited remodeling (A2–C2), FTSG with extensive remodeling (A3–C3), an example of a region with dense connective tissue (A4–C4), and a region with loose connective tissue (LCT; A5–C5). The first column shows panels with eosin and hematoxylin (HTX) staining (Panel A), the second column shows HABP staining, where hyaluronic acid is stained brown (Panel B), and the third column shows Masson Trichrome staining (TCM), collagen is blue, elastin and keratin are red, and hair is yellow (Panel C). The epidermis of the host is indicated with ‘Sk’ where applicable. Black arrowheads indicate sebaceous glands (rows 1, 2, and 3). Long black arrows indicate hair follicles (rows 1, 2, and 3). A region of LCT with high cellularity is indicated by a short black arrow (bottom row). In the second (A2–C2) and third (A3–C3) rows, host skin and FTSG are in continuum and delineated by the dotted line. Bars A–C = 100 µm. Abbreviation: HABP, HA-binding probe.

**Figure 2.**
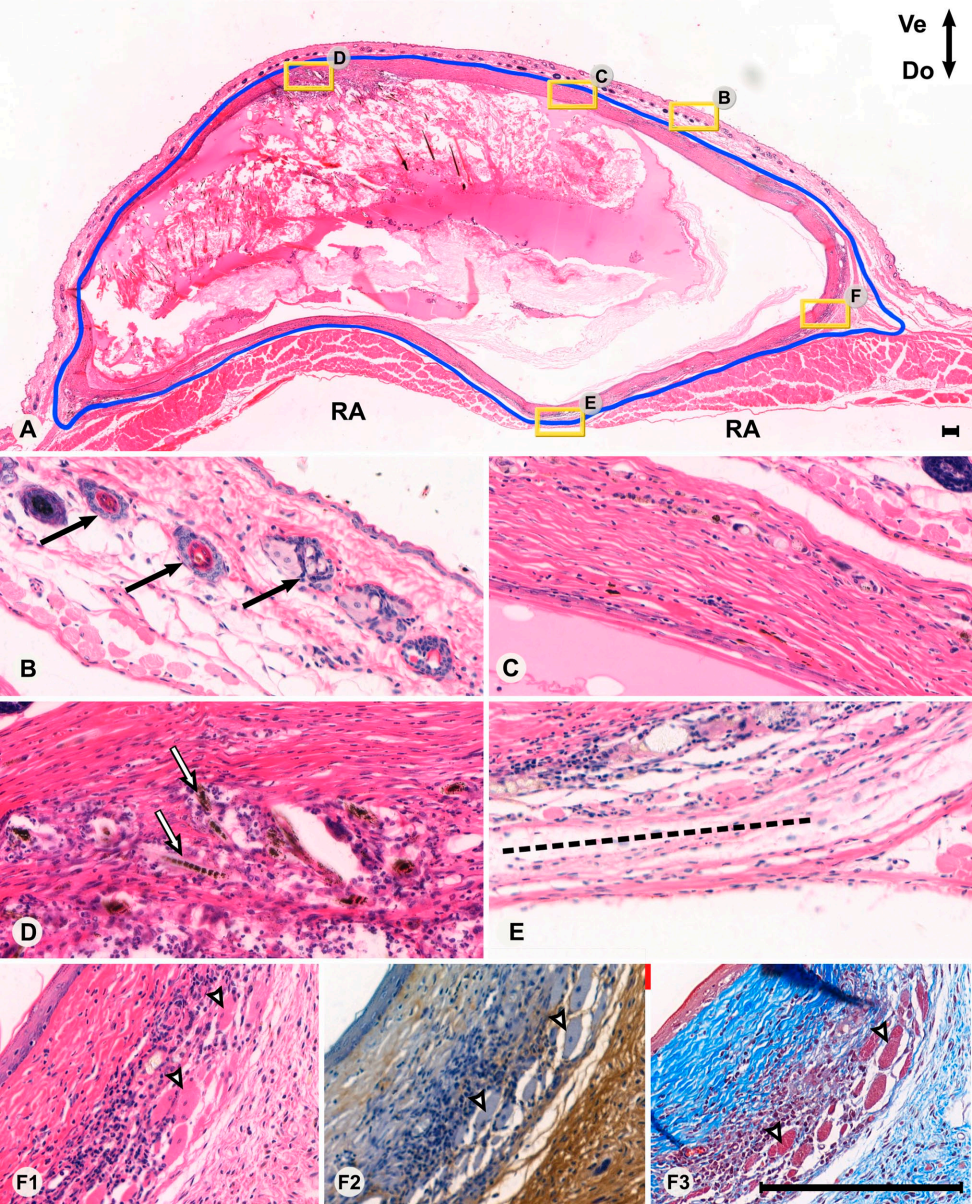
Overview (Panel A) and high-magnification views (Panels B–F3) of a section from a mouse with the full-thickness skin graft in the onlay position (outlined in blue). Areas of high-magnification views are marked with a yellow frame with corresponding letters as in the lower panels. In the top panel, the ventral orientation is facing up and dorsal orientation is facing down. The striated muscles of the RA are indicated in Panel A. The section is delimited by the host skin ventrally (Panel B) and the peritoneal wall dorsally (Panel E), where the border between the graft and linea alba (dotted line in Panel E) can be seen. Normal hair follicles of the host skin are indicated in Panel B (black arrows). A view of the graft with mostly dense connective tissue is demonstrated in Panel C. A view of the graft in a cellular region of loose connective tissue is demonstrated in Panel D, also displaying remnants of hair follicles and hair (white arrows). A corner of the graft is indicated in Panels F1–F3, showing an eosin and hematoxylin (HTX) staining (F1), HA-binding probe (F2), and Masson Trichrome (F3), respectively. Panels F1–F3 display different types of connective tissue in proximity to various levels of cellularity, presence of collagen, and presence of hyaluronan. Degenerating muscle fibers in the graft are indicated with white arrows. Bars A–F = 200 µm. Note that due to different magnification, the scale bar in the top panel’s lower right corner is relatively small. All remaining panels (B–F) have identical magnification. Abbreviation: RA, rectus abdominis.

**Figure 3.**
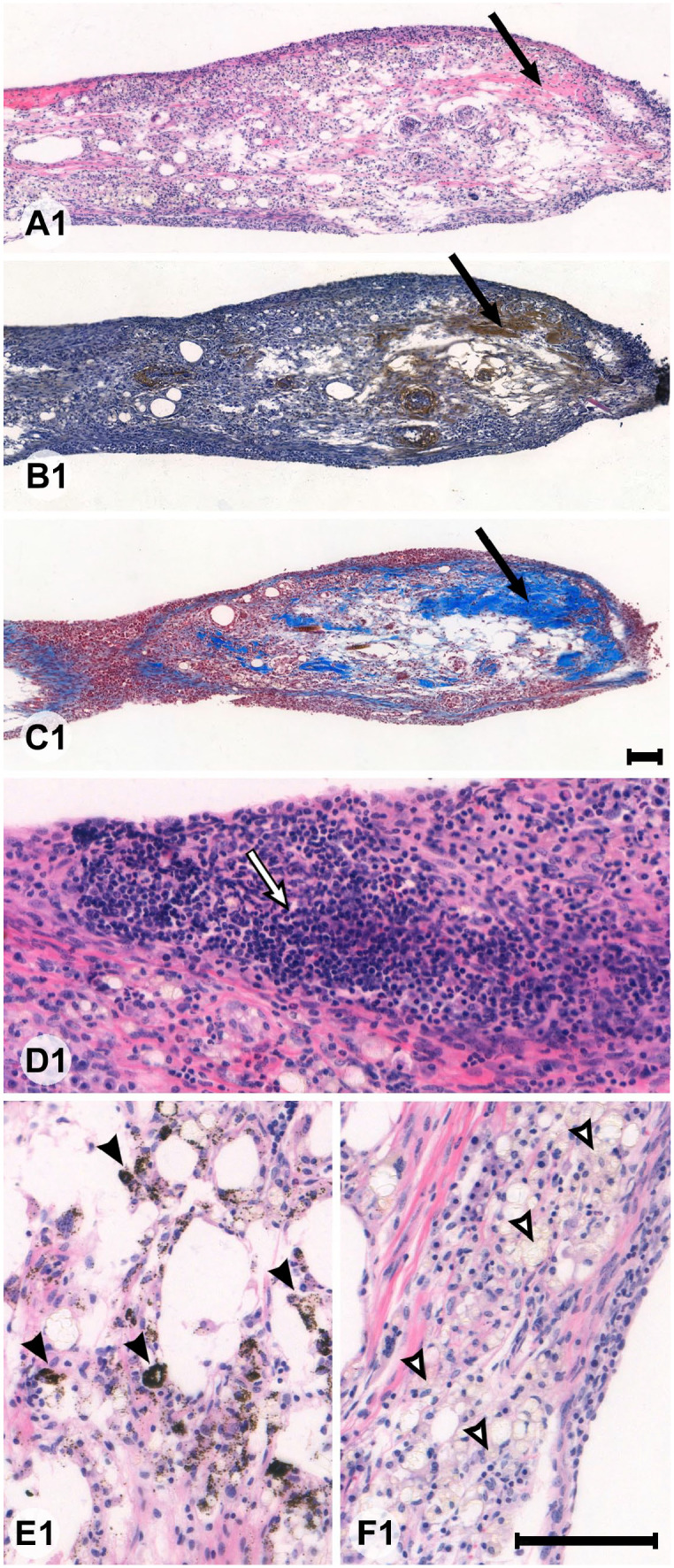
Sections of a full-thickness skin graft from an animal where the graft had failed to integrate. Panels A1–C1 are low-magnification views of the same area with eosin and hematoxylin (HTX) (A1) staining, HA-binding probe (B1) staining, and Masson Trichrome (C1) staining. The remaining panels (D1–F1) are high-magnification views of areas of the same graft. Only a miniscule proportion of the graft had developed dense connective tissue (long black arrow in A1–C1). Large parts of the graft were made up of dense groups of inflammatory monocytes (white arrow in D1), vacuoles surrounded by phagocytized melanin (black arrows in E1), or macrophage-like cells reminiscent of foam cells (white arrows in F1). Bars A1–F1 = 100 µm but note the different lengths of the scale bars due to the use of two magnifications.

Despite the mice being sacrificed at the same time, there were marked differences in appearance of the FTSGs (Fig. 1). All skin grafts showed some signs of remodeling. In the most mature cases, hair, hair follicles, and sebaceous glands had all disappeared entirely and become replaced with extensive, relatively cell-sparse, DCT. In other cases, hair follicles and glands were in different states of remodeling (Fig. 1). Despite various states of remodeling, two different types of connective tissue were present and recognizable in 8 of 10 FTSGs—one characterized by DCT with fewer cells, seemingly derived from the deeper parts of dermis (row 4, Fig. 1), and one characterized by loose connective tissue (LCT) with high cell density (row 5, Fig. 1). In the latter, intact or degenerating hair follicles and sebaceous glands (Fig. 2) were present, but much lesser organized connective tissue. In Masson Trichrome staining, the DCT was intensively stained blue (collagen) whereas LCT had weaker staining. In general, the more voluminous the DCT, the more organization (with clear orientation of collagen and cells) was present. Integration into the host abdominal wall was most visible in the corners of the graft (Fig. 2) and generally had a more integrated appearance in mice operated with the onlay method. In one mouse, the FTSG differed dramatically in appearance (Fig. 3). It was somewhat smaller in CSA than the other grafts, contained very little DCT, and in eosin and HTX staining appeared to contain many lymphocytes and phagocytic macrophages. Circular cell arrangements, suggestive of remnants of former hair follicles and cystic cavities, were much more frequent than in other grafts. Furthermore, this graft showed a clean separation from the enclosed cavity within the host animal, with no sign of tissue integration. This specimen was interpreted as a “failed” graft of the 20 studied, that for some reason had degenerated after implantation. Staining indicated low presence of both collagen and HA, and it was excluded from further grouped analysis in the study, for example, when calculating mean CSA.

On cross sections of the anterior abdominal wall, the area of the grafts varied between 2.3 and 11.7 mm^2^ (mean 4.25 ± 2.20 mm^2^). The two smallest grafts (2.4 and 2.3 mm^2^) were either grafts with little tissue remodeling or where the graft itself had failed (see above). The largest graft (11.7 mm^2^) stood out from the rest due to a high degree of both inflammation, remodeling, and angiogenesis together with extensive signs of integration along the edges of the graft. As mentioned above, prominent areas of DCT were present in a majority of specimens (15 out of 20). In the failed specimen, it accounted for approximately 5% of the CSA whereas in the remaining 19 specimens, DCT accounted for 52.2 ± 22.6% of the CSA. In general, those specimens where the proportion of DCT was highest were those where the loss of hair and hair follicles was most complete. Organization of the LCT was generally more dubious, clear borders between actual LCT and degenerating remnants of hair follicles or groups of inflammatory cells meant that a precise estimate of the proportion of LCT was not possible in all specimens. For this reason, further analyses focused on the DCT.

### Histochemical Staining for HA and Collagen

In the host’s skin, regardless of whether that skin was directly overlying the FTSG or situated on the anterolateral abdominal wall beside the area of implantation, the dermis was uniformly stained brown with HABP (indicating the rich presence of HA) and moderately blue with Masson trichrome (TCM) (indicating the presence of collagen fibrils; Fig. 1). In the FTSGs, the patterns of HABP and TCM staining were more variable. In FTSGs showing less remodeling (row 2, Fig. 1), HABP staining and TCM staining were both present with similar intensity. In FTSGs with more extensive remodeling, intense blue TCM staining was not necessarily associated with intense brown HABP staining. Instead, HA staining in DCT areas in the same animal varied considerably, usually in bundle-like structures. HA staining was generally absent in LCT tissue (Fig. 1). HABP staining in the DCT was rated from (–) indicating no HABP staining to (++) indicating HABP staining in >75% of the DCT. Three raters (coauthors A.E.T., A.G., and U.H.) analyzed 16 specimens and the inter-rater reliability was deemed to be good (Fleiss’ kappa 0.683; *p*<0.001). However, HABP ratings did not differ between specimens implanted with the IPOM or onlay method (*p*<0.114) and in the total study group, HABP grade did not correlate with the total graft area (*p*<0.511), proportion of DCT (*p*<0.171), or any other variable studied (data not shown).

### Collagen Evaluation (CHP and Masson Trichrome Staining)

**Figure 4.**
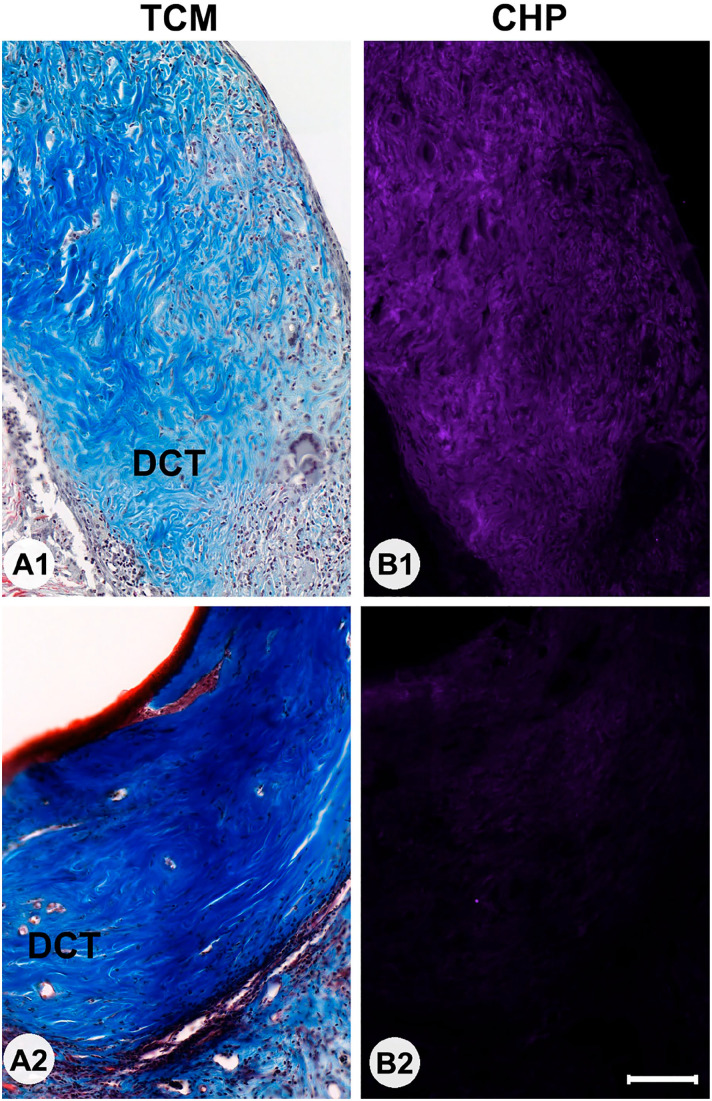
Sections of grafts stained with Masson Trichrome (TCM; Panels A1 and A2) or collagen hybridizing peptide (Panels B1 and B2) in two animals with markedly different fluorescence collagen hybridizing peptide (CHP) staining intensities. In both instances, the rich presence of collagens (blue) can be seen in the dense connective tissue (DCT; shown in the panels). In one instance, CHP intensity is high (B1) suggesting the presence of unfolded collagen helices, whereas in the other (B2) CHP intensity is low, suggesting a DCT with less active remodeling. Bars A and B = 100 µm.

Masson Trichrome staining was used to locate areas with collagen within the section. Most CHP-positive staining was seen in the DCT areas of the grafts. In all mice, CHP intensity was low in the overlying skin of the host animal. There was a notable variation in CHP staining intensity of the graft between specimens (Fig. 4), with the lowest two specimens displaying intensity units of 2.3 and 5.8 above background staining intensity whereas the highest two specimens displayed staining intensity units of 82.9 and 84.0 above background staining intensity, respectively.

Quantification of CHP intensity in the grafts showed a non-significant (*p*<0.097) but notable difference in intensity between the IPOM (47.5 ± 30.0) grafts and the onlay (22.5 ± 21.1) grafts.

### Immunofluorescence Cell Markers

**Figure 5.**
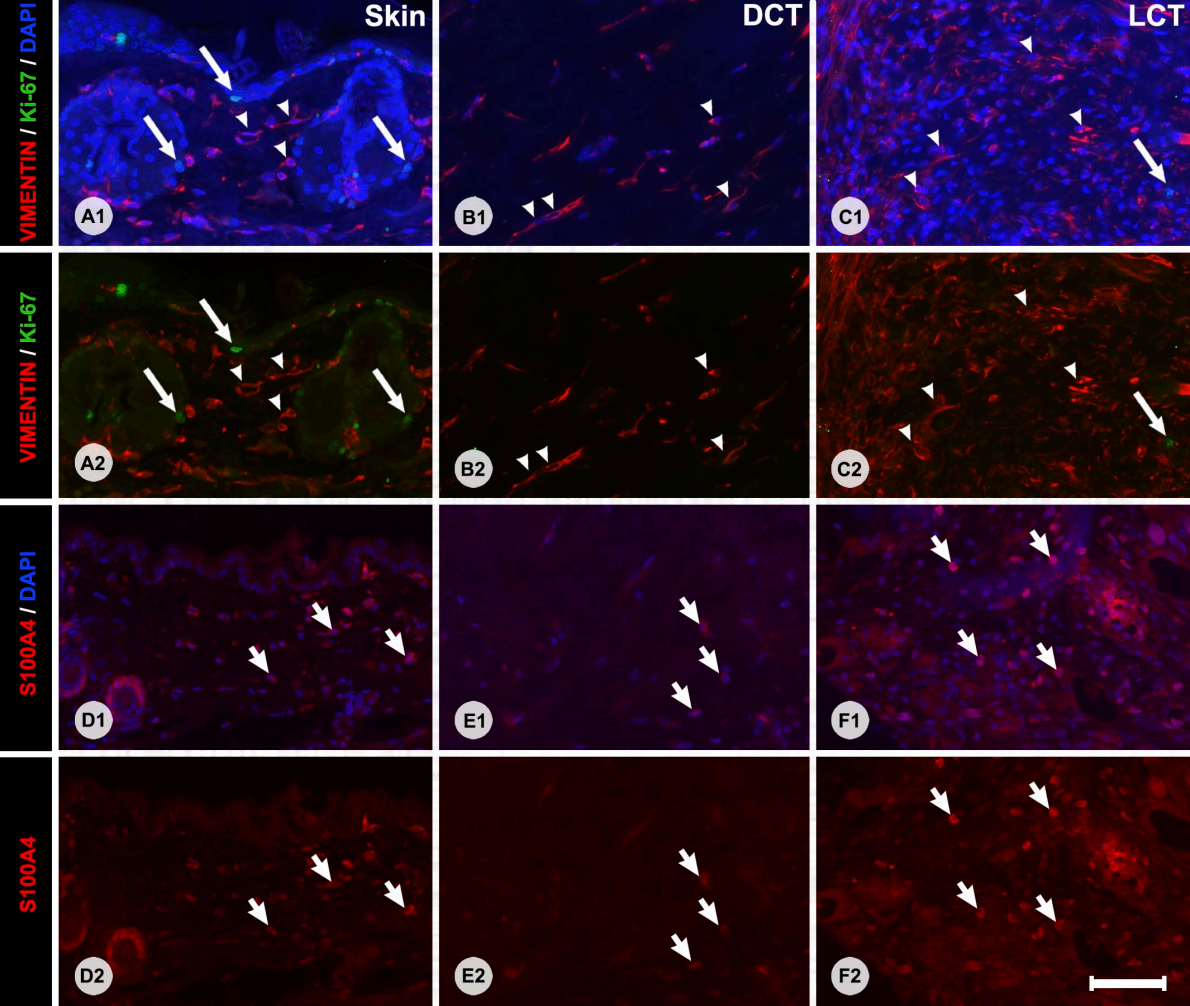
Immunofluorescence staining with primary antibodies against vimentin and Ki-67 (Panels A1–C2) or S100A4 (Panels D1–F2). Panels A1–F1 show overlapping 4′,6-diamidino-2-phenylindole (DAPI) counterstain to visualize nuclei, whereas Panels A2–F2 show the images without DAPI counterstain. Panels on the left show labeling patterns in the skin of the host, middle panels show labeling patterns of DCT areas, and the panels on the right show the labeling patterns of LCT. Vimentin-positive cells (white arrows), S100A4-positive cells (white short arrows), and vimentin-negative/Ki-67-positive cells (long white arrows) are shown in each panel. Please note that DAPI labeling is dimmer in the S100A4 panels, this is due to the alkaline pre-treatment of the slides necessary for the S100A4 primary antibody. Bars A–F = 50 µm. Abbreviations: DCT, dense connective tissue; LCT, loose connective tissue.

**Figure 6.**
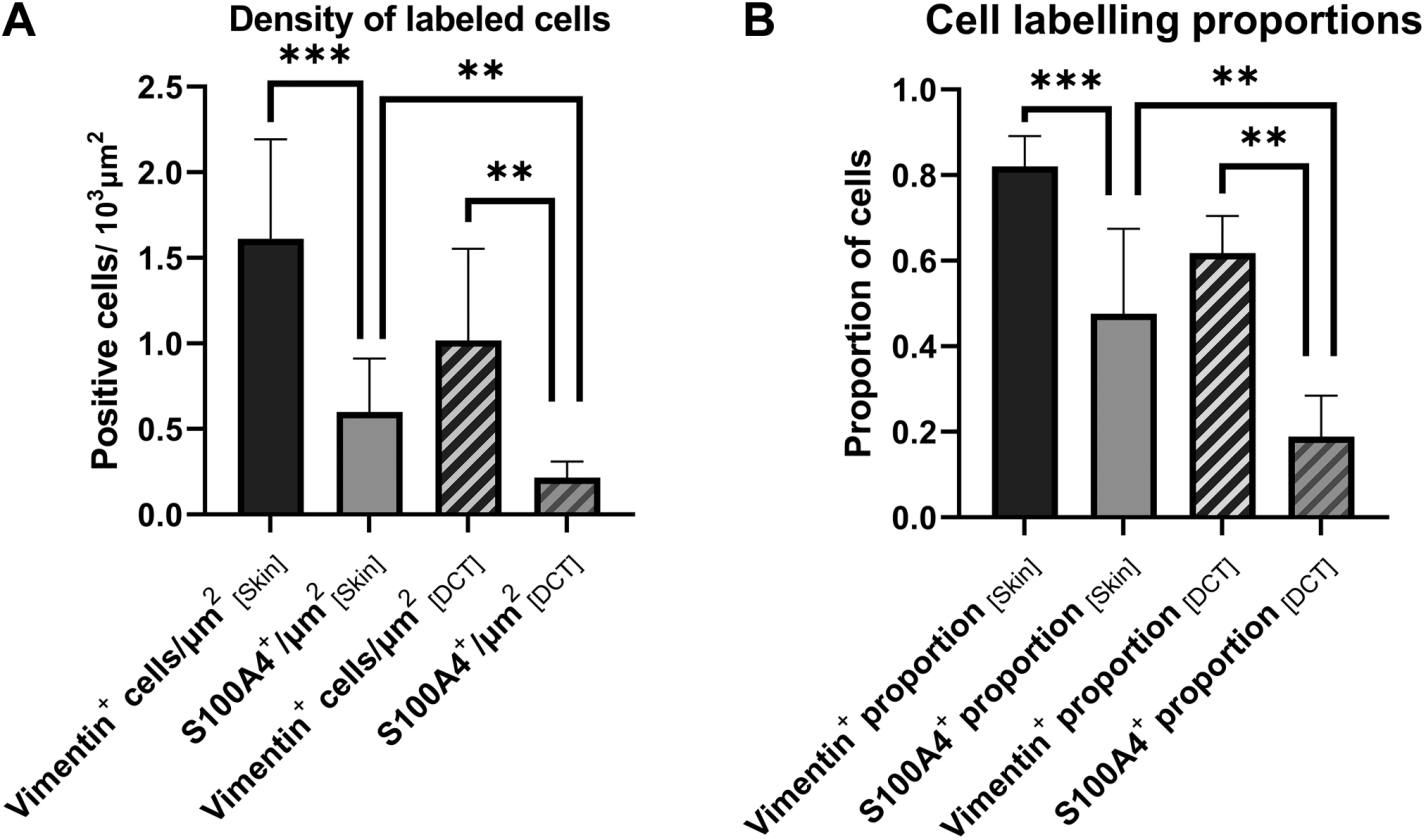
Bar graph displaying the number of labeled cells per 1000 µm^2^ (Panel A) and the proportion of cells (Panel B) positive for vimentin and S100A4 in host skin and DCT of the graft, respectively. Asterisks indicate significant pairwise differences between estimates, tested with Wilcoxon signed-rank test. Error bars indicate 95% CI. Abbreviations: DCT, dense connective tissue; CI, confidence interval.

Vimentin-positive cells were widespread in the dermis and deep subcutaneous layers of the host skin, and generally had a stellate or elongated shape (Fig. 5). These cells were identified as typical fibroblasts. Vimentin-positive cells were also sporadically encountered in association with striated muscle fibers close to the FTSG, but these were ignored because activated muscle satellite cells are also known to express vimentin.^17^ In our samples, the morphology of vimentin-positive cells differed between graft locations and specimens. In FTSGs with little remodeling, vimentin labeling was more diffuse showing less densely packed fibroblasts. In LCT areas usually containing hair follicle remnants, there were dense clusters of overlapping vimentin-positive cells, making precise quantification or characterization difficult or impossible. On the other hand, vimentin-positive cells were easy to identify and quantify in host skin dermis and in the DCT areas of the grafts. Most cells in both skin dermis and DCT areas that were quantifiable were vimentin-positive (Fig. 5). Dermal endothelial cells and pericytes of capillaries have also been reported to express vimentin.^19,20^ Capillaries were sparse but present to some degree in all FTSG samples. Given that some tangential sectioning of capillary walls is unavoidable, and that *bona fide* fibroblast is also likely to be found close to capillaries, we also quantified vimentin-positive cells in association with capillaries. This means that our quantitative data likely include some vimentin-positive pericytes and endocytes besides classic fibroblasts, but given that capillaries made up a minor portion of the sections the skewness in data is suggested to be of minor importance for the overall results. For all specimens, the mean percentage of vimentin-positive cells in the dermis of host skin was 81.9 ± 12.2% and 61.7 ± 13.8% in the DCT areas of the graft (Fig. 6). The corresponding densities of vimentin-positive cells were 1.61 ± 1.01 cells/1000 µm^2^ in host skin and 1.02 ± 0.84 cells/1000 µm^2^ in the DCT areas of the graft (Fig. 6). Neither percentage (*p*<0.441) nor density (*p*<0.141) of vimentin-positive cells in the DCT areas of the graft differed between the IPOM and onlay positions.

In both host skin and FTSGs, S100A4-positive cells were significantly fewer than vimentin-positive cells (Fig. 6) in both highly differentiated grafts and in those less differentiated. For all specimens, the mean percentage of S100A4-positive cells was 50.0 ± 26.7% in the dermis of host skin and 18.8 ± 14.3% in the DCT areas of the grafts. The corresponding densities of S100A4-positive cells were 0.61 ± 0.45 cells/1000 µm^2^ in host skin and 0.22 ± 0.14 cells/1000 µm^2^ in the DCT areas. Neither percentage (*p*<0.187) nor density (*p*<0.696) of S100A4-positive cells in the DCT areas differed between IPOM and onlay positions.

A subset of nine specimens was double-stained for vimentin and Ki-67 (Fig. 5). Ki-67-positive cells were found in all specimens but not always in the DCT areas. Between 0 and 10 positive cells (median 6) were found in the DCT areas of each full cross section and the majority of these were also vimentin positive. However, most Ki-67-positive cells were found in areas of the graft other than the DCT. Ki-67-positive cells were frequently seen in the LCT areas but were not always double-stained for vimentin. Ki-67-positive cells were also seen in the skin of the host but seldom as double-stained vimentin-/Ki-67-positive cells, usually in the epithelia or the hair follicles (Fig. 5).

All specimens were also stained for α-smooth muscle actin. Generally, this antibody labeled cells throughout the dermis and graft in approximate correspondence with the vimentin staining described above. In addition, it also labeled the cytoplasm in glandular structures of the dermis and epithelia. This, in addition to a quite variable labeling intensity, made a reliable and informative quantification of smooth muscle actin cells difficult. Therefore, no further quantification was carried out with this marker.

### Correlations

No significant correlations were seen between the total graft CSA and any other variable. However, the proportion of graft that had developed into DCT was positively correlated to the proportion (*p*<0.033, ρ = 0.673) but not density (*p*<0.651, ρ = 0.164) of vimentin-positive cells within the DCT. Interestingly, there was a negative correlation between the proportion of S100A4-positive cells in the DCT and the density of vimentin-positive cells (*p*<0.023, ρ = −0.705) indicating that grafts with fewer vimentin-positive cells had a higher proportion of S100A4-positive cells. The proportions of S100A4-positive cells in DCT areas and host skin were positively correlated to the intensity of CHP staining (*p*<0.029, ρ = 0.720 and *p*<0.050, ρ = 0.667, respectively). A review of all correlations tested is found in Appendix Table A1.

## Discussion

In this study, considerable morphological changes were seen following transplantation of FTSG into the abdominal wall in a murine model. The main findings of the study were as follows: (1) Most grafts undergo remodeling from typical full-thickness skin into two coexisting types of connective tissue, characterized by either fewer fibroblasts and a voluminous ECM (denoted DCT in this article) or by a sparse ECM and densely packed cells (denoted LCT); (2) vimentin-positive and S100A4-positive cells were found throughout DCT areas of the graft, but S100A4-positive cells were less common than vimentin-positive cells; (3) the proportion of vimentin-positive cells in DCT areas correlated with the proportion of the graft that had changed to DCT; (4) the proportion of S100A4-positive cells in DCT areas correlated with a greater degree of ECM remodeling, as indicated by CHP intensity; (5) grafts in the IPOM and onlay positions underwent similar remodeling in terms of cell composition and ECM at the selected time of sacrifice; and (6) grafts occasionally fail to form DCT.

To our knowledge, this murine model is the only one that has followed the long-term outcome of FTSG implantation in the two typical hernia repair positions. Previous clinical studies have shown the validity, efficacy, and relative safety of FTSG implantation, but few have studied the long-term fate of these grafts. The present study on mice, as well as previous investigations, shows that transplanted skin undergoes extensive remodeling in the new anatomical site, whether it be in the onlay or IPOM position. Interestingly, epithelial skin cells are still present 8 weeks after implantation in this model, and they remain facing inward in the graft. The epidermis of the mouse is only a few cells thick, and selective removal of the epidermis before implantation would be technically challenging. However, it would be interesting to follow the remodeling process in a skin graft with the epidermis removed and see if this influences the formation of DCT within the graft. The current study does not include data on the mechanical characteristics of the individual grafts at the time of sacrifice. Therefore, the precise correlation between DCT formation and functional properties of the graft remains unknown. However, given that the ECM is the most structurally important component of the dermis, and responsible for the incredible tensile strength of skin,^21^ there should be a considerable correlation between the formation of DCT in the graft and its ultimate mechanical strength.

In this study, we chose to study fibroblasts using two common markers, vimentin and S100A4. Vimentin-deficient mice have a markedly reduced wound repair response with negative impact on keratinocyte mobilization and differentiation myofibroblast activation and collagen deposition, suggesting that vimentin serves a crucial role in tissue remodeling.^10^

In this study, grafts with the highest proportion of DCT in relation to the total area showed a high fraction (but not density) of vimentin-positive cells within the DCT areas. Furthermore, 13–67% of cells in DCT areas were vimentin positive with a morphology suggestive of mesenchymal cells. However, recent studies on classic fibroblast markers (such as vimentin and S100A4) have revealed that immune cells may also express these markers under certain conditions, and also regulate collagen turnover.^22^ Also, our IgG isotype control staining showed that a small proportion of cells (<2%) could be mislabeled by nonspecific IgG binding. Therefore, a minor portion the quantified cells can be expected to be of non-fibroblast origin. Moreover, given that the proportion rather than the density of vimentin-positive cells seems to influence the degree of remodeling into DCT, other vimentin-negative cells in the graft could have had an inhibitory influence in the formation of DCT in the graft. This would explain the inverse relationship between vimentin- and S100A4-positive cells observed in the DCT areas. This relationship, however, did not appear in the host skin where proportions and densities of S100A4-positive and vimentin-positive cells were similar. Further studies are needed to see whether this difference between host skin and graft is due to the presence of other types of vimentin-positive cells in the graft or due to less S100A4 protein serving as a signaling molecule. An important question is whether grafts characterized by a high proportion of S100A4-positive cells with a high density of CHP staining represent a different pattern of remodeling or that this is simply an earlier stage in the remodeling process. S100A4 is interesting as this calcium-binding signaling protein seems to play an important dynamic role in fibroblast activation. This is exemplified in Senolt et al.,^23^ where increases in matrix metalloproteinase 1, 2 and 9 protein and gene expression levels were seen in response to S100A4 oligomer treatment of synovial fibroblasts derived from patients with rheumatoid arthritis. In a previous study from our group, it was observed that these MMPs were not associated with collagen in FTSGs, but we cannot rule out the involvement of other MMPs.^24^ Perhaps specimens with high CHP density and S100A4 levels represent grafts that are still in an active phase of collagen degradation and remodeling. Some grafts with low CHP density had a high collagen level (Fig. 3) indicating the presence of mature collagen. CHP staining targets all types of unfolded triple-helix collagen, revealing areas where collagen has been cleaved by proteolytic enzymes. CHP density in the graft varied between mice, and with position, being somewhat higher in the IPOM grafts than in the onlay grafts. One interpretation could be that grafts in the onlay position reach a more mature state earlier than grafts in the IPOM position. Grafts in the onlay position have previously been shown to maintain a closer overall proximity to the connective tissue of the recipient animal^7^ with a larger surface area available for the diffusion of cytokines and metabolites. These grafts were implanted in both positions and animals were sacrificed at the same time, so the position of surgical application of the graft would seem to be associated with different remodeling processes possible due to skin graft interaction between tissues with different cell types and compositions based on graft location. In terms of ECM remodeling and deposition, this is an intricate process involving both active cell-mediated assembly through integrins and other membrane-bound factors and passive processes (in the case of helical assemblies of collagens and laminins).^25^ Although recent work on the present FTSG mouse model has shown that many common collagens and MMPs are present in the graft,^24^ the precise ECM make-up, including collagen composition and integrin expression in relation to other connective tissues, remains to be elucidated.

Over the years, fibroblast has become understood to exhibit more variation and lineages that previously thought. Fibroblasts do not only display tissue-specific expression profiles but also considerable variation within tissues.^26,27^ In skin, reticular and papillary fibroblasts have been detected and characterized, with evidently various reactive characteristics. In a mouse wound-healing model, the ectoderm-derived superficial fibroblasts were shown to mount a more proliferative regenerative response whereas the mesoderm-derived deeper fibroblasts mounted a response characterized by protein synthesis and wound migration signaling.^8^ Furthermore, a rich and significant variation in steady-state expression profiles seems to modulate local tissue characteristics in different tissues and is likely to also modulate different responses to perturbations, meaning that some fibroblast subtypes might be more vital for graft integration than others.^27^ This may explain the morphological differences between grafts in different implantation positions with the involvement of different tissue layers. Interestingly, whereas the current study concerns FTSG taken from the back of mice that did not include the underlying fascia, a recent study showed that fascia-derived fibroblasts and matrix are crucial in healing of deep skin wounds and that wound remodeling in superficial and deep skin wounds follows very different trajectories, with the later resulting in a more dramatic remodeling driven by fascia-derived fibroblasts.^28^ As discussed earlier, the grafts placed in the onlay position could be interpreted as reaching a more mature morphology faster. It is noteworthy that the onlay position creates a large area of interface between the disturbed host fascia and the transplanted FTSG. Thus it remains to be studied what role the receiving fascia could play in the integration of the FTSG.

No significant correlation was seen between HABP staining and characteristics of the specimens, despite a significant and relatively good (Fleiss’ kappa: 0.683) inter-rater agreement between examiners. Our original hypothesis was that HABP intensity would be high in mature grafts with a high proportion of DCT or in DCT areas with ongoing remodeling, due to the interplay between HA and the wound-healing response. No such correlations were seen, possibly due to the method for estimating the presence of HA or that the phase of remodeling with the highest dynamic expression of HA occurs earlier than the observations made in this study.

The graft that lacked integration and remodeling was unexpected because we have not observed such failure in previous studies using the same murine model.^7^ The pronounced inflammation present in that graft suggests that possibly failure was due to transplant rejection. Notably, graft rejection has not been a problem in previous studies because graft recipient and donor both came from a line of C57BL/6 mice that had been backcrossed at least 10 generations and were practically genetically identical. Theoretically, but not very likely, a spontaneous mutation in an Human leukocyte antigen (HLA) gene or other immunoregulatory gene may have occurred, causing mismatch between donor and recipient and thus rejection. Still, there are other mechanisms that could cause a graft to fail, such as wound/graft contamination, infection, unexpected thrombosis, or other technical issues that might have passed unnoticed. Clinically, we have seen that a few patients operated with an autologous graft in the IPOM position have developed a seroma. Future animal studies should be designed to investigate the limitations of FTSG methods and to see if intraoperative precautions, such as graft “rifting,” could reduce or eliminate the risk of seroma formation.

As far as the clinical relevance of this study is concerned, the present results suggest that implantation of FTSG in the IPOM position shows a tissue remodeling behavior similar to grafts implanted in the more established onlay position. This encourages ongoing and future trials to evaluate the advantages of IPOM FTSG compared with synthetic implant in complicated hernia repair. The apparent slower integration process with active remodeling at the time of sacrifice after 8 weeks of implantation in the IPOM position suggests a longer period of abdominal wall load restriction would be advisable.

## Appendix

**Table A1.** Correlations between quantitative variables.

Variables	Proportion of DCT to Total Area	Vimentin+_skin_ Dens	Vimentin+_dct_ Dens	S100a4+_skin_ Dens	S100a4+_dct_ Dens	Vimentin+_skin_ [%]	Vimentin+_dct_ [%]	S100a4+_skin_ [%]	S100a4+_dct_ [%]	chp Intensity
Proportion of DCT to total area	N/A	*p*<0.948 ρ = 0.021	*p*<0.651 ρ = 0.164	*p*<0.898 ρ = 0.050	*p*<0.881 ρ = −0.059	*p*<0.557 ρ = 0.189	*p*<**0.033** ρ = **0.673**	*p*<0.651 ρ = −0.176	*p*<0.651 ρ = −0.176	*p*<0.681 ρ = 0.126
Vimentin+_skin_ Dens	*p*<0.829 ρ = 0.070	N/A	*p*<**0.011** ρ = **0.699**	*p*<0.670 ρ = −0.145	*p*<0.374 ρ = 0.316	*p*<0.122 ρ = 0.433	*p*<0.513 ρ = 0.210	*p*<0.312 ρ = −0.336	*p*<0.476 ρ = −0.255	*p*<0.236 ρ = −0.371
Vimentin+_DCT_ Dens	*p*<0.651 ρ = 0.164	*p*<**0.011** ρ = **0.699**	N/A	*p*<0.798 ρ = −0.100	*p*<0.713 ρ = 0.134	*p*<0.846 ρ = 0.063	*p*<0.779 ρ = 0.091	*p*<**0.050** ρ = −**0.677**	*p*<**0.023** ρ = −**0.705**	*p*<0.214 ρ = −0.430
S100A4+_skin_ Dens	*p*<0.898 ρ = 0.050	*p*<0.670 ρ = −0.145	*p*<0.798 ρ = −0.100	N/A	*p*<0.831 ρ = −0.083	*p*<0.670 ρ = 0.145	*p*<0.088 ρ = 0.600	*p*<**0.003** ρ = **0.800**	*p*<1.00 ρ = 0.00	*p*<0.546 ρ = 0.233
S100A4+_DCT_ Dens	*p*<0.881 ρ = −0.059	*p*<0.374 ρ = 0.316	*p*<0.713 ρ = 0.134	*p*<0.831 ρ = −0.083	N/A	*p*<0.815 ρ = 0.085	*p*<0.206 ρ = 0.438	*p*<0.637 ρ = 0.183	*p*<**0.043** ρ = **0.616**	*p*<0.932 ρ = 0.033
Vimentin+_skin_ [%]	*p*<0.557 ρ = 0.189	*p*<0.122 ρ = 0.433	*p*<0.846 ρ = 0.063	*p*<0.670 ρ = 0.145	*p*<0.815 ρ = 0.085	N/A	*p*<0.090 ρ = 0.510	*p*<0.433 ρ = 0.264	*p*<0.894 ρ = 0.049	*p*<0.484 ρ = −0.224
Vimentin+_DCT_ [%]	*p*<**0.033** ρ = **0.673**	*p*<0.513 ρ = 0.210	*p*<0.779 ρ = 0.091	*p*<0.088 ρ = 0.600	*p*<0.206 ρ = 0.438	*p*<0.090 ρ = 0.510	N/A	*p*<0.488 ρ = 0.267	*p*<0.920 ρ = 0.036	*p*<0.054 ρ = −0.624
S100A4+_skin_ [%]	*p*<0.0381 ρ = −0.333	*p*<0.312 ρ = −0.336	*p*<**0.050** ρ = −**0.677**	*p*<**0.003** ρ = **0.800**	*p*<0.637 ρ = 0.183	*p*<0.433 ρ = 0.264	*p*<0.488 ρ = 0.267	N/A	*p*<0.058 ρ = 0.650	*p*<**0.050** ρ = **0.667**
S100A4+_DCT_ [%]	*p*<0.651 ρ = −0.176	*p*<0.476 ρ = −0.255	*p*<**0.023** ρ = −**0.705**	*p*<1.00 ρ = 0.00	*p*<**0.043** ρ = **0.616**	*p*<0.894 ρ = 0.049	*p*<0.920 ρ = 0.036	*p*<0.058 ρ = 0.650	N/A	*p*<**0.029** ρ = **0.720**
CHP intensity	*p*<0.681 ρ = 0.126	*p*<0.236 ρ = −0.371	*p*<0.214 ρ = −0.430	*p*<0.546 ρ = 0.233	*p*<0.932 ρ = 0.033	*p*<0.484 ρ = −0.224	*p*<0.054 ρ = −0.624	*p*<**0.050** ρ = **0.667**	*p*<**0.029** ρ = **0.720**	N/A

Bivariate correlational analyses for the quantitative measurements were outlined in the Results section. Significant correlations are marked in bold. Dens indicates the estimated number of cells/1000 µm^2^, [%] indicates the percentage of cells positive for the marker, with the total number of cells as the denominator. Skin indicates the host animal skin on the same section as the graft. DCT indicates the DCT region of the graft. Abbreviations: DCT, dense connective tissue; CHP, collagen hybridizing peptide.
